# Dietary Fatty Acids Change Circulating Fatty Acids, Microbial Putrefactive Postbiotics and Betaine Status in the Cat

**DOI:** 10.3390/ani10122310

**Published:** 2020-12-06

**Authors:** Dennis E. Jewell, Matthew I. Jackson

**Affiliations:** 1 Department of Grain Science and Industry, Kansas State University, Manhattan, KS 66506, USA; 2 Hill’s Pet Nutrition, Topeka, KS 66601, USA; matthew_jackson@hillspet.com

**Keywords:** arachidonic acid, DHA, EPA

## Abstract

**Simple Summary:**

The cat is an obligate carnivore that is well adapted to dietary polyunsaturated fatty acids (PUFA), perhaps because of the variance resulting from normal consumption of organ meat which is high in PUFA, and storage lipid which is often relatively low in PUFA. Although able to tolerate and thrive with this variation, cats have a metabolic response to fatty acids that is relatively unknown. This study shows that dietary PUFA resulted in changing circulating concentrations of that specific PUFA. Increasing dietary eicosapentaenoic acid EPA and docosahexaenoic acid DHA (E&D) resulted in little change in total circulating PUFA as compared to increasing dietary arachidonic acid (ARA) which resulted in an increased concentration of total circulating PUFA. Cats responded to increased dietary E&D by reducing circulating cholesterol as compared to control fed cats. Increasing dietary PUFA also resulted in a decrease in circulating betaine, dimethylglycine and sarcosine in comparison to the cats consuming the control food at the end of the study. Changing dietary PUFA also changed circulating concentrations of gut microbial purification postbiotics. Increasing dietary ARA resulted in an increased concentration of indoleacetate, indolepropionate and indoleacetylglutamine in comparison to cats fed foods enhanced with increased E&D. Increasing E&D resulted in a decreased concentration of 4-ethylphenylsulfate, 3-methyl catechol sulfate and 4-vinylphenol sulfate at the end of the feeding period as compared to cats fed increased ARA or fed the unsupplemented control food. These changes suggest that support of single carbon metabolism would benefit cats with increasing dietary PUFA, that increasing E&D beneficially lowered cholesterol and that dietary PUFA influenced gut microbes resulting in changes in their postbiotics.

**Abstract:**

There is a normal variation of polyunsaturated fatty acids (PUFA) in the foods consumed both by the domestic cat and wild felines. This variation may lead to specific changes in metabolites and circulating fatty acids that influence health and response to disease. Therefore, in order to evaluate the response to these changes in dietary PUFA three foods were formulated: a complete and balanced control food (COF) with no enhanced source of added PUFA (ARA = 0.08%, EPA & DHA = 0.01%), Test food 1 (E&DF) like the COF with added eicosapentaenoic acid EPA and docosahexaenoic acid DHA (E&D = 0.36%)) from menhaden fish oil, and Test Food 2 (ARAF) like the COF with added arachidonic acid (ARA = 0.16%) from liver. All test foods had similar protein concentrations and similar vitamin and mineral concentrations while the PUFA supplemented foods had slightly higher fat concentrations. Cats (*n* = 36) were fed a pre-trial food for 28 days and then assigned to a group fed either the control, E&DF or ARAF for 56 days (12 cats per group). Blood samples were drawn and serum analyzed for fatty acids, albumin, urea, creatinine, cholesterol and triglycerides at the beginning of the study and after consuming the test foods for 28 and 56 days. Plasma was similarly analyzed for metabolomics. Increasing dietary E&D resulted in reduced cholesterol, betaine, dimethyl glycine, sarcosine and 4-ethylphenylsulfate. Increasing dietary ARA resulted in reduced betaine, dimethyl glycine and sarcosine and an increased concentration of indoleacetate, indolepropionate and indoleacetylglutamine. These data suggest a benefit of dietary single carbon metabolism support for cats supplemented with ARA or E&D. Moreover, the reduction in circulating cholesterol and triglycerides through dietary E&D supplementation could benefit cats with hyperlipidemia. Further research into the interrelationship between dietary PUFA and the gut microbe will benefit from the data showing that ARA increased specific positive postbiotics (i.e., indoleacetate, indolepropionate) while E&D supplementation showed the benefit of reducing some postbiotics which have been associated with reduced health (4-ethylphenylsulfate, 3-methyl catechol sulfate and 4-vinylphenol sulfate).

## 1. Introduction

Dietary changes in polyunsaturated fatty acids (PUFA) through enhanced foods or supplements are a means to alter the metabolic milieu in a way that enhances health and disease resistance [1]. It is known that changing dietary PUFA in the cat can influence the immune response through changes in leukotriene (LTB_5_) and lymphocyte populations and proliferations, with eicosapentaenoic acid EPA and docosahexaenoic acid DHA (E&D) having a greater effect than alpha linolenic acid [2]. This reduction in immune response with E&D is similar to that seen in the dog where reduced cell mediated immune response was reported [3]. This is also similar to the decreased inflammatory immune response in the presence of dietary E&D in swine and poultry [4] and the immune system modulation in humans [5]. However, changes in dietary PUFA do not only change immune response but also alter levels of metabolites that influence health and disease.

The microbiome is known to influence its host through metabolites which it produces (postbiotics) and it has been shown that this influence responds to dietary lipids in the cat [6]. These postbiotics can be absorbed in the colon and into the host circulation of cats, mice and humans [6,7,8]. The consumption of E&D has been shown to change the gut microbiome through altering the abundance of microbiota which are known to be involved with specific physiological conditions [9,10]. It is reasonable that the microbiota milieu changes in response to available substrate. Moreover, postbiotics shift not only with changing microbiota but through a shift in metabolism in the microbiota present [7]. Microbial postbiotics derived from the putrefaction of phenylalanine and tyrosine, or tryptophan, result in production of putrefactive phenols, or indoles, which are absorbed by the host [11]. In order to detoxify and excrete these postbiotics the host may conjugate them to sulfate or amino acids (e.g., glutamine) [12]. These postbiotics and sulfated postbiotics may have a deleterious effect on health, especially regarding inflammation and renal disease [13,14,15], or a positive influence such as enhanced energy utilization or gastrointestinal function [16,17]. This study is unique in that to our knowledge these changes have not been evaluated in a study simultaneously changing ARA and E&D in cats.

The metabolic milieu associated with changing microbial function may also influence specific biochemical pathways. This may be especially important in cats as they exhibit metabolic eccentricities with regards to one-carbon metabolism, amino acids and vitamins, which has been proposed to arise in part from a diet rich in fat [18]. The cat has increased requirements for protein and higher endogenous losses than other mammals (e.g., dog, rat, human as expressed as gm/kcal intake [18]). The one-carbon metabolism pathway of cats has been reviewed [18] with the cat being unique in its production of the amino acid felininine, decreased biosynthesis of taurine, and increased methionine adenosyltransferase activity perhaps driving an increased need for N,N,N-trimethylglycine (hereafter, betaine). Similar to the reduction in immune response produced by E&D supplementation, betaine has anti-inflammatory actions [19]. Betaine also restores gut barrier protein expression that has been reduced due to exposure to inflammatory bacterial metabolites [20]. Furthering the parallels between PUFA and betaine, dietary DHA and ARA PUFA improve [21,22], while n-6 corn oil-derived PUFA reduce, barrier integrity [23]. Improved gut barrier protein expression bolsters the intestinal ‘firewall’, which may influence the accessibility of microbial postbiotics to portal resorption into host circulation [24]. Surprisingly, given the parallels between PUFA and betaine with regards to inflammation (e.g., increased taurine in response to betaine or reduced LTB_5_ in response to increased E&D) or gut microbiome and barrier function, there is a paucity of research on the influence of PUFA on betaine and one carbon metabolism. It has been reported [25] that betaine supplementation changes lipid metabolism. Although it is not clear in cats, a response in circulating betaine and subsequent one carbon metabolites (dimethylglycine and sarcosine) may be hypothesized by the influence of n-3 PUFA on 5-methyl tetrahydrofolate and betaine homocysteine methyltransferase [26].

This study evaluated the effect of changing dietary ARA, E&D on circulating lipids, postbiotics and one carbon metabolites in cats with a focus on understanding how these changes differed between increased dietary ARA or E&D.

## 2. Materials and Methods

The study protocol was reviewed and approved by the Institutional Animal Care and Use Committee, Hill’s Pet Nutrition, Inc., Topeka, KS, USA (permit #FP518.1.1.0 -A-F-D-ADH- MULTI-120 -MULTI). All cats were cared for by animal care research technicians who were masked to the group identity during sample collection. Moreover, all sample analyses were completed by technicians who were masked to group identity of the cats associated with the sample. All cats were domestic short hair breed and owned by the commercial funders. During the 28-day washout period, all cats were fed the same pre-trial complete and balanced feline maintenance food. Cats were then assigned to either control or one of the two treatment foods (which were all complete and balanced for adult cats as described by the Association of American Feed Control Officials) and consumed that food exclusively for 84 days. The COF has no specific source of E&D or ARA and analyzed below detectable limits for E&D and 0.08% ARA. Test food 1 (E&DF) was the COF plus 0.23% EPA and 0.12% DHA from menhaden fish oil. Test food 2 (ARAF) was the COF plus 0.08% ARA from chicken liver.

Twenty-four neutered male and 12 spayed females with and average age of 8.4 years (Std. Dev 1.5 years) were assigned so that an equal gender ratio and similar age was used for each treatment. All cats were group housed and had access to toys, an enclosed porch with natural lighting, and water ad libitum. Food of an amount controlled to maintain body weight was offered for 20 h per day. Thirty-six cats were assigned to foods; 12 to COF, and 12 to each of the treatment foods. One cat was removed from the group eating E&DF for non-food related reasons. On the evening before blood collection, access to food was blocked and returned after phlebotomy. Therefore, blood samples were collected after approximately 12 h of fasting. Blood samples were collected (8 mL) by jugular venous puncture under anesthesia (within 10 min of 2 mg/kg telazol IM) and serum and plasma separated by centrifugation at 30,000× *g* for 10 min. Global metabolomics was measured before starting test foods and after 84 days of eating one of the test foods. Clinical blood chemistry and circulating fatty acids were completed at the times of metabolomic analysis and after 56 days of eating one of the test foods. A COBAS c501 module (Roche Diagnostics Corporaion, Indianapolis, IN, USA) was used for clinical blood chemistry analysis and Metabolon (Morrisville, NC, USA) performed the metabolomics analysis of the plasma as previously described [6,27]. In short, the samples were processed with a proprietary methanol based solvent extract. These were than split and either used for derivatization and gas chromatography-mass spectrometry of used in the underivatized form and analyzed by liquid chromatography-mass spectrometry. Samples were analyzed in a randomized order. The data for each analyte were normalized using the median value for each run-day block (this minimized the interday instrument drift while maintaining the intraday variability in the samples). If a specific analyte was below the detection limit for the specific compound and instrumentation then, when present, values were imputed with the observed minimum for that particular compound. Block normalization was completed before imputed values were added. Values in Table 5 are the means of the ratios of the initial values divided by final values (the first three columns reporting change within a group) or ratios of the mean values at the end of the study (the last three columns reporting change between groups).

Fatty acid (FA) composition and other nutrients of the experimental foods were determined by a commercial laboratory (Eurofins Scientific, Inc., Des Moines, IA, USA). Proximate analyses were completed using the following techniques: moisture-AOAC 930.15; protein-AOAC 2001.11; fat-AOAC 954.02; fiber-AOAC 962.09; and ash-AOAC 942.0; Circulating plasma fatty acids were determined using gas chromatography of the FA esters. The sum of dietary saturated fatty acids (SFA) was determined as follows: 8:0 + 10:0 + 11:0 + 12:0 + 14:0 + 15:0 + 16:0 + 17:0 + 18:0 + 20:0 + 22:0 + 24:0. The sum of dietary monounsaturated fatty acids (MUFA) was determined as follows: 14:1 + 15:1 + 16:1 + 17:1 + 18:1 + 20:1 + 22:1 + 24:1. The sum of dietary PUFA was determined as follows: (18:2 × (n-6)) + (18:3 × (n-6)) + (18:3 × (n-3)) + (18:4 × (n-3)) + (20:2 × (n-6)) + (20:3 × (n-6)) + (20:3 × (n-3)) + (20:4 × (n-6)) + (20:4 × (n-3)) + (20:5 × (n-3)) + (21:5 × (n-3)) + (22:2 × (n-6)) + (22:4 × (n-6)) + (22:5 × (n-6)) + (22:5 × (n-3)) + (22:6 × (n-3)) as previously described [28].

In order to compare treatment effects for body weight, serum biochemical profiles and fatty acid composition, SAS 9.4 Proc Mixed was used (SAS Institute, Cary, NC, USA). Time and treatment (based on food consumed) were used as independent variables and individual cat identification as a repeated measure. Normality was assessed by evaluation of the residual plot and the use of the Kolmogorov–Smirnov test. A *p* value ≤ 0.05 was used as a cut off for significance for evaluations at each time point and for treatment effect on the change during the feeding period. Post-hoc analysis was completed using the PDIFF option in Proc Mixed. For the analysis of the metabolomics data, change over time was evaluated using day zero for each cat as its own control. For the difference between treatment groups at the end of the study each individual cat was used as an independent measure. All metabolomics data were log-transformed before analysis of significance was completed because of a significant number of non-normal analytes. A *p* value < 0.05 and a false discovery rate correction q value <0.1 were used as cut offs for significance.

## 3. Results

The food ingredient mix and analytical results are shown in Table 1. As designed the foods are similar with the exception of increased EPA (0.23% vs. <0.01), DHA (0.13% vs. 0.01) in E&DF and increased arachidonic acid in ARAF (0.16% vs. 0.08%) with both test foods compared to the COF. These increased fatty acids came with a slight increase in total fat in both E&DF (15.06% vs. 14.67%) and ARAF (16.01 vs. 14.67%) as compared to the COF.

There was no change in body weight and all treatment means stayed within colony normal for circulating concentrations of: albumin, total protein, urea nitrogen, creatinine, trigylcerides and cholesterol. There was no effect (*p* = 0.72) of dietary lipid source on food intake with consumption of control, E&DF and ARAF resulting in 86, 83 And 83 Kcal/kg^¾^ power, respectively. There were two analytes that responded to treatment. Urea was lower in both groups of cats eating E&DF and ARAF as compared to the cats eating the COF while cholesterol was lower in the cats eating E&DF as compared to those eating the COF or ARAF (Table 2).

As expected, increasing dietary concentrations of E&D as well as ARA resulted in increasing circulating concentration of these fatty acids (Table 3). The 0.08% increase in dietary ARA had a greater effect on circulating ARA (an increase of circulating ARA of 9.6 ± 1.3 mg/dL) as compared to that of EPA with a 0.23% increase in dietary concentration (an increase in circulating EPA of 4.9 ± 0.4 mg/dL) or DHA with a 0.12% increased dietary concentration (an increase of circulating DHA of 4.2 ± 0.4 mg/dL). The increase in circulating DHA and EPA concentration was approximately similar despite dietary EPA increasing twice as much as dietary DHA increased in E&DF. There was also a different influence of these PUFA on total circulating PUFA with E&DF having a greater reduction in total PUFA than either control or ARAF. This difference was the result of E&DF consumption resulting in reduced linoleic (LA) and ARA concentrations as compared to controls or those cats eating ARAF. These changes happened while linolenic acid (αLA) remained unchanged. Moreover, there was a difference in the n-6 to n-3 ratio in the circulating fatty acids with E&DF ending in a lower ratio than the cats eating either control or ARAF which were not different from each other (Table 3).

Cats eating E&DF had reduced saturated fatty acid concentrations during the study as compared to either cats eating COF or cats eating ARAF which were not different from each other. Cats eating E&DF also had a lower change in monounsaturated fatty acids as compared to both the control and ARAF treatment groups, the latter of which were not different than each other. These results of the fatty acid classes were mostly driven by changes in stearic and palmitic saturated fatty acids and palmitoleic and oleic monounsaturated fatty acids (Table 4).

The non-targeted metabolomics analysis detected 642 metabolites. Of this total, the analytes that changed over time (*p* < 0.05, *q* < 0.1) in at least one treatment and were different between treatments at the end of the study (*p* < 0.05, *q* < 0.1) are shown in Table 5. There were 133 metabolites that met these criteria with 94 of those being lipids. As expected, the fatty acids that were specifically supplemented (E&D and ARA) increased in this analysis as well as many analytes showing subsequent inclusion into phospholipids, complex lipids and oxidized lipid metabolites. Two acyl carnitines were elevated in both groups consuming E&DF and ARAF (myristoylcarnitine and cerotoylcarnitine) while two were elevated only in the group consuming ARAF (arachidonoylcarnitine and adrenoylcarnitine). Many glycerophosphatidylcholines (GPC) changed in response to dietary fatty acids; many GPC in cats consuming E&DF decreased during the feeding period while these GPC in the control group and ARAF either did not change or increased. This was also seen in glycerophosphatidylethanolamine (GPE), phosphatidylinositol (GPI) and sphingomyelin metabolites. TDhe DHA containing phospholipids (1-stearoyl -2-docosahexaenoyl-GPC, 1-oleoyl-2-docosahexaenoyl-GPC and 1-palmitoyl-2- docosahexaenoyl –GPC) were elevated after consuming E&DF. Moreover, stearoyl sphingomyelin was increased after consumption of E&DF and ARAF. Indoleacetate, indolepropionate, and indoleacetylglutamine are tryptophan metabolites that are exclusively contributed by bacteria metabolism and all were lower in the cats eating E&DF at the end of the study when compared to those consuming ARAF. There were also reductions in the cats eating E&DF of the postbiotics phenylacetylglutamate, phenol sulfate, 4-methoxyphenol sulfate, 2-hydroxyphenylacetate, 3-methyl catechol sulfate, 4-ethylphenylsulfate and 4-vinylphenol sulfate at the end of the study when compared cats consuming ARAF. Relative to control fed cats at the end of the study, E&DF-fed cats manifested reduced levels of 4-methoxyphenol sulfate, 3-methyl catechol sulfate, 4-methylcatechol sulfate, 4-ethylphenylsulfate, 4-vinylphenol sulfate and p-cresol sulfate as well as the glutamate, glutamine, glycine and serine conjugates of phenylacetate. There was a reduction in a significant number of postbiotics in E&DF group relative to both ARAF and the controls group. At the end of the study all measured postbiotics were numerically lower in the E&DF group as compared to controls with 4-methoxyphenol sulfate, phenylacetylglutamate, phenylacetylglutamine, phenylacetylglycine, phenylacetylserine, 3-methyl catechol sulfate, 4-methylcatechol sulfate, 4-ethylphenylsulfate, 4-vinylphenol sulfate, p-cresol sulfate, being significantly reduced. The single carbon pathway metabolites betaine, dimethylglycine and sarcosine all were lower in the cats eating E&DF and ARAF when compared to those cats eating the COF.

## 4. Discussion

In a previous study, it was reported that there was a significant compositional shift in circulating EPA and DHA as a percent of total fatty acids when these lipids were consumed by cats [29]. With this increased intake (1.126 g EPA and 0.624 g DHA per day in a capsule) circulating EPA as a percentage of total plasma fatty acids increased approximately 10 fold and percentage DHA tripled with no change in ARA, this resulted in an 83% reduction in the circulating ARA/(E&D) ratio. This can be compared to the absolute value (mg/dL) response seen with the addition of dietary EPA and DHA in this study; EPA concentration increased more than 10 fold and DHA concentration more than tripled and ARA concentration decreased by 20%. This comparison is possible as similar ratios are calculated when these concentrations are expressed as a change in the ARA/(E&D) percentage ratio (as there were only small changes in measured total nonesterified fatty acids). Together, these changes in EPA, DHA and ARA led to the observed 81% reduction in circulating ARA/(E&D) ratio which was observed in the E&D supplemented cats when compared to baseline which was similar to the changes observed when compared to the control fed cats at the end of the study. The circulating concentration of ARA initially exceeds E&D; therefore, the response to the increased dietary ARA resulted in a smaller (6%) increase in the ARA/(E&D) ratio when compared to the initial value of cats before eating ARAF as compared to the 81% comparative reduction resulting from the changes in the initially lower E&D concentration when dietary E&D were increased. The changes in the E&D supplemented cats [29] as a ratio to total nonesterified fatty acids was 1.6% to 9%, which was similar to this study which expressed as a ratio to total fatty acids was a move from 1.5 to 8%. Therefore, it is likely that this amount of E&D would also not negatively affect platelet aggregation in cats, which can be a health concern [29]. The change in the circulating ARA/(E&D) ratio in cats in the current study supplemented with E&D resulted in a circulating ratio for ARA/(E&D) of 1.7 which was similar to the ratios of 1.1Tab–1.2 for ARA/(E&D) which were shown to suppress inflammation (e.g., skin thickness change in response to histamine) and some immune responses (reduced CD21+ B cells, total T cells and T helper cells) as reported in cats [2]. The changes in the ratios of 3.4 to 0.6 [29] are comparable to this study, which in response to E&D supplementation changed from 9 to 1.7. These higher ratios in our study are likely the result of less consumed E&D and higher circulating concentrations of ARA (again likely a reflection of intake). A 13 mg/dL concentration increase in the combined fatty acids of ARA, EPA and DHA (all three fatty acids were supplemented together) resulted in a reduction in the risk for oxalate stone formation in cats [30]. This can be compared to the 9.6 mg/dL increase in either ARA or E&D combined in this study. However, as improvement in the risk of oxalate stone formation in cats fed the combined fatty acids was with the increased consumption of ARA and E&D combined, these observed changes in fatty acids by themselves do not show if either of the dietary changes in this study would be sufficient to improve the risk of stone formation. The results showing that circulating SFA were increased with ARA supplementation while circulating MUFA, linoleic, and oleic acids were decreased with E&D supplementation indicate that there is an overall influence of these dietary changes on the fatty acid milieu. However, these changes are of unknown physiological significance and the basis for further investigations.

It is not surprising that dietary changes in ARA, E&D would change their respective circulating concentrations and be associated with changes in the concentrations of their specific fatty acid moieties. Changes in the fatty acid composition of glycosylphosphatidylinositols, phosphatidylethanolamines and sphingomyelins were both expected and observed. The reduction in cholesterol in response to dietary E&D is similar to what we have shown previously [6] so this seems to be a repeatable finding in cats which appears to be different than that seen in humans [31,32]. This suggests that supplementary dietary E&D could be of value for a food designed to aid in the management of feline hypercholesterolemia. The current study demonstrated an effect of changing dietary fatty acids on the microbiome produced postbiotics, with E&D consumption being associated with a reduced concentration of several sulfate and amino acid conjugates of putrefactive phenols when compared to the ARA-supplemented and non-supplemented control cats at the end of the feeding period. Furthermore, putrefactive postbiotics of the indole class were reduced by E&D feeding relative to ARA-fed cats, but not compared to control cats. This (with the exception of 4-vinylphenol sulfate) was not the result of a reduction during the time of E&D consumption but rather because the concentration change was significantly lower than the change in the control group. Our previous study detected changes in circulating microbiome putrefactive postbiotics of the phenol and indole classes, however that study utilized DHA-enriched fish oil (DHA:EPA ratio = 7.3) and cats were fed for only 4 weeks rather than the DHA:EPA ratio in the current study of 0.56 with a 12 week feeding period [6]. The changes in phenols in that study included both decreases (3) and increases (4) in response to the increased PUFA, and increases in five indoles including indoleacetate and indolepropionate; none of these changed significantly in the current study which may indicate nuance in the responsiveness of the gut microbiome to EPA versus DHA or that length of feeding results in differential conditioning of the microbiome. However, in contrast to the effects of E&D observed here, the addition of ARA resulted in an increased concentration of several phenolic and indolic putrefactive postbiotics. Feeding corn oil, a source of n-6 PUFA, in the context of a high-fat diet was shown in mice to reduce expression of proteins critical to maintenance of gut barrier integrity [23]. Dietary ARA and EPA have been shown to be incorporated into intestinal phospholipids in a dose and time dependent manner [33]; the length of feeding in the current study was long enough to see the maximal increase in intestinal incorporation previously observed in piglets [33]. Intriguingly, in that study [33], it was also observed that EPA incorporation into intestinal phospholipids constituted only about 50% of the incorporation of ARA, despite identical dose and timing regimens. In the current study, increased ARA appearance in serum was greater than increases in E&D despite the latter being fed at higher dietary levels. It has been shown [34] that dietary supplementation with the beneficial microbial product butyrate decreased fecal levels of ARA and that fecal ARA, a proxy of ARA incorporation into intestinal epithelial phospholipids, was positively associated with inflammatory cytokines. It has also been reported to be inversely associated with the beneficial bacteria *Akkermansia* [34]. It has also been shown [35] that dietary supplementation with ARA decreased beneficial gut microbes and butyrate in male but not female mice. A comparison of the effects of EPA and ARA PUFA on cultured intestinal cells (CaCo-2) has been made; although ARA increased ICAM-1 protein expression associated with leukocyte recruitment in inflammatory bowel disease and increased MCP-1 which leads to macrophage infiltration, EPA had no such effects [36]. A previous study showed that when dietary ARA (0.14%) was combined with DHA (0.14%) the overall increased effect, rather than reducing gut barrier integrity, was a reduced intestinal permeability and villae length to improve gut barrier integrity. However, in that study in rats the individual effects of these fatty acids were not assessed and there was co-presence of pre and probiotics [21]. Additionally, fish oil provision was shown to increase gut barrier integrity in humans [22]. Taken altogether, previous results and the data from this study may indicate that changes to gut barrier may at least partially underpin the increased appearance of putrefactive postbiotics in the cats fed ARA but not E&D. Given that many of the postbiotics found at lower levels after E&D supplementation compared to ARA supplementation can be detrimental to (e.g., renal) health in humans [13,15], it may be that selection of the type of PUFA in dietary formulations influences the degree to which health benefits may be realized through the gut microbiome.

Perhaps the most striking result of the metabolomics data as influenced by dietary changes in ARA and E&D is the change in the single carbon metabolism pathway. In both supplemented groups, there was a significantly lower concentration of betaine, dimethyl glycine and sarcosine as compared to the control fed cats at the end of the study period. This complete reduction of the single carbon pathway suggests that it is, in the cat, a pathway wide effect. In the literature, betaine supplementation has been used as the modifier of this pathway. For example, in humans it has been observed [37] that there was an increase in betaine when EPA and DHA-containing krill oil was fed; however, the krill oil used in that study was 50% in glycerophosphatidylcholine form, which is a highly bioavailable source of choline readily converted to betaine [37]. The effect of E&D on single carbon metabolism could be part of the reason that fish oil consumption was shown to be associated with increased homocysteine concentration in humans [38]. That study also showed that there was no apparent impediment to homocysteine conversion to cysteine, leading to the conclusion that remethylation of homocysteine was likely reduced. Although those authors excluded changes in folate or vitamin B-12 as contributory to increased homocysteine, betaine levels were apparently not considered. Contrarily, a comparison of the effects of n-3 (EPA, DHA) and n-6 (ARA) supplementation in mice showed that n-3 fatty acids decreased, while n-6 fatty acids increased, circulating homocysteine concentrations. They also showed that n-3 and n-6 provision differentially impacted expression of enzymes which determine the fate of homocysteine (remethylation versus transsulfuration). Betaine supplementation has been shown to increase muscle EPA, DHA and total n-3 fatty acids as well as reducing the (n-6):(n-3) ratio of muscle in freshwater shrimp without changing muscle ARA or total n-6 fatty acid levels [39]. The same study showed that crude fat levels of muscle were decreased by betaine supplementation and total fatty acids were unchanged, indicating that there may be a specific effect of betaine on n-3 PUFA [39]. Further supporting a physiological interaction of betaine with PUFA, betaine supplementation decreased abdominal fat but quadratically increased muscle fat content in a betaine dose-dependent manner in lambs [40]. The same group showed that betaine increased muscle PUFA and monounsaturated fatty acid content while concurrently decreasing saturated fatty acid levels. Adding additional weight to the proposal that betaine metabolism is tightly linked to fat metabolism, it has also been shown that betaine supplementation reduced specific depot and total fat mass in both chow-fed and high fat diet-fed mice, and that further betaine reduced proliferation of cultured adipocytes [25]. This same report demonstrated that betaine supplementation also decreased circulating triglycerides, total cholesterol and low-density lipoprotein, which indicates a systemic effect on fat partitioning [25]. However, as these data were not generated in cats their application to feline nutrition requires more research. Betaine influences the incorporation of PUFA into brain phospholipids in piglets [41] where phospholipid forms of DHA and ARA were increased with dietary betaine. However, this study showed that hepatic phospholipid forms of these fatty acids were unchanged. Showing that the betaine-PUFA relationship has implications for antioxidant defense, it has been previously reported that high levels of n-3 PUFA feeding accompanied by ethanol intake can decrease antioxidant enzyme activity, but that betaine co-provision ameliorates this detrimental effect [42]; the percent of energy provided by dietary n-3 PUFA in the current study (1.3%) was an order of magnitude less than that previously reported (13.8%) by Varatharajalu et al., [43]. The work by Goldstein et al. (1994) may provide a mechanistic underpinning to our observation that PUFA reduced circulating betaine [44]. Their group showed that betaine transport by an erythrocyte betaine transporter was inhibited by ARA; although EPA and DHA were not examined, they showed that the potency of inhibition of betaine transport was directly correlated to the number of unsaturations present in a given fatty acid such that the increasing order of potency of inhibition was oleic, linoleic, arachidonic [44]. In the current study, we show that both E&D (n-3) and ARA (n-6) intakes were associated with a decline in circulating betaine in the domestic cat, an obligate carnivore. Although previous reports described above show unambiguously that betaine supplementation impacts fat partitioning by increasing muscle PUFA levels while decreasing adipose fat stores we were unable to find any published reports indicating that PUFA supplementation influences betaine metabolism. Our data may provide an impetus to supplement betaine into feline diets that are rich in PUFA.

A limitation of this study was that the sources for ARA and E&D were liver and fish oil respectively. Although these ingredients provide the fatty acids in a fashion that adds the desired concentration and allows the planned intake, they also contain other fatty acids and nutrients which might influence the responses measured. Conversely, the use of commercially available ingredients indicates that translating these findings into real-world benefits may be feasible.

## 5. Conclusions

Dietary fatty acids EPA and DHA as well as ARA influence circulating metabolites through uptake and incorporation into fatty acid containing molecules such as the fatty acid moieties on glycerol phosphoethanolamine and glycerol phosphocholine. Dietary EPA and DHA decrease while ARA increases levels of circulating microbial putrefactive postbiotics 3-methyl catechol sulfate, 4-ethylphenylsulfate and 4-vinylphenol sulfate. Both dietary EPA and DHA as well as ARA decreased circulating betaine, dimethyl glycine, and sarcosine.

## Figures and Tables

**Table 1:** Food composition and ingredient make up of pre-trial and test foods (grams/100 g as mixed or as fed, unless otherwise stated).

Ingredient or Analyte	Pre-Trial Food	Control	E&D Food	ARA Food
Rice	15.9	39.6	39.6	40.2
Corn gluten meal	13.9	24.7	24.7	20.8
Poultry by-product meal	26.1	19.7	19.7	16
Corn	24.6	0	0	0
Pork Fat	13.9	9.8	8.4	9
Palatability Enhancer	1.3	1.4	1.4	1.4
Menhaden Fish oil	0	0	1.4	0
Chicken livers, hydrolyzed, dry	0	0	0	7.5
Lactic acid (84% lactic acid)	1.8	1.2	1.2	1.2
Choline Chloride	0.7	0.7	0.7	0.7
Methionine	0.5	0.2	0.2	0.2
Taurine	0.1	0.1	0.1	0.1
Minerals and Vitamins	1.2 ^a^	2.6 ^b^	2.6 ^c^	2.9 ^d^
Moisture	5.77	6.55	6.56	7.04
Protein	31.99	34.2	33.82	33.91
Fat	20.69	14.67	15.06	16.01
Atwater Energy ^€^ (kcal/kg)	4121	3799	3821	3880
Ash	5.23	4.87	4.77	4.67
Crude Fiber	0.8	1	1	NA
Calcium	0.96	0.67	0.68	0.69
Phosphorus	0.82	0.67	0.63	0.64
Sodium	0.34	0.31	0.29	0.31
Capric acid [10:0]	0.02	0.01	0.01	0.01
Lauric acid [12:0]	0.02	0.01	0.01	0.01
Myristic acid [14:0]	0.2	0.14	0.22	0.14
Palmitic acid [16:0]	4.19	3.01	2.97	3.14
Palmitoleic acid [16:1]	0.57	0.37	0.44	0.38
Steric acid [18:0]	2.01	1.48	1.38	1.62
Oleic acid [18:1]	6.87	4.80	4.55	4.90
Arachidic acid [20:0]	0.04	0.03	0.03	0.03
LA [18:2 (n-6)]	3.17	2.52	2.41	2.51
aLA [18:3 (n-3)]	0.13	0.12	0.13	0.11
ARA [20:4 (n-6)]	0.09	0.08	0.09	0.16
EPA [20:5 (n-3)]	<0.01	<0.01	0.23	<0.01
DHA [22:6 (n-3)]	0.01	0.01	0.13	0.02
SFA ^£^	6.32	5.12	4.71	5.39
MUFA ^¥^	7.61	5.29	5.76	5.4
PUFA ^π^	3.39	2.87	3.23	2.96
(n-6) FA ^Ω^	3.43	2.72	2.62	2.8
(n-3) FA ^θ^	0.16	0.15	0.61	0.16
(n-6):(n-3) ratio	21.4	18.1	4.3	17.5

NA—not available because of analytical error, predicted value is 0.9%. ^a^ Added minerals and vitamins: calcium (0.126), potassium (0.291), sodium (0.081), chloride (0.409), magnesium (64 mg/kg), iron (87 mg/kg), copper (9 mg/kg), manganese (9 mg/kg), zinc (180 mg/kg), iodine (2.1 mg/kg), selenium (0.24 mg/kg), vitamin A (3232 IU/kg), vitamin C (14mg/kg), vitamin D (646 IU/kg), vitamin E (825 IU/kg), thiamine (54 mg/kg), riboflavin (13 mg/kg), pantothenic acid (21 mg/kg), niacin (189 mg/kg), pyridoxine (24 mg/kg), folic acid (3 mg/kg), biotin (0.3 mg/kg) vitamin B_12_ (0.13 mg/kg). ^b^ Added minerals and vitamins: calcium (0.171), potassium (0.540), sodium (0.084), chloride (0.561), magnesium (64 mg/kg), iron (87 mg/kg), copper (9 mg/kg), manganese (9 mg/kg), zinc (180 mg/kg), iodine (2.1 mg/kg), selenium (0.24 mg/kg), vitamin A (3570 IU/kg), vitamin C (10 mg/kg), vitamin D (714 IU/kg), vitamin E (1115 IU/kg), thiamine (60 mg/kg), riboflavin (14 mg/kg), pantothenic acid (23 mg/kg), niacin (209 mg/kg), pyridoxine (27 mg/kg), folic acid (3 mg/kg), biotin (0.3 mg/kg) vitamin B_12_ (0.14 mg/kg). ^c^ Added minerals and vitamins: calcium (0.171), potassium (0.540), sodium (0.084), chloride (0.561), magnesium (64 mg/kg), iron (87 mg/kg), copper (9 mg/kg), manganese (9 mg/kg), zinc (180 mg/kg), iodine (2.1 mg/kg), selenium (0.24 mg/kg), vitamin A (3570 IU/kg), vitamin C (10 mg/kg), vitamin D (714 IU/kg), vitamin E (1115 IU/kg), thiamine (60 mg/kg), riboflavin (14 mg/kg), pantothenic acid (23 mg/kg), niacin (209 mg/kg), pyridoxine (27 mg/kg), folic acid (3 mg/kg), biotin (0.3 mg/kg) vitamin B_12_ (0.14 mg/kg). ^d^ Added minerals and vitamins: calcium (0.281), potassium (0.426), sodium (0.084), chloride (0.450), magnesium (64 mg/kg), iron (87 mg/kg), copper (9 mg/kg), manganese (9 mg/kg), zinc (180 mg/kg), iodine (2.1 mg/kg), selenium (0.24 mg/kg), vitamin A (3570 IU/kg), vitamin C (10 mg/kg), vitamin D (714 IU/kg), vitamin E (1115 IU/kg), thiamine (60 mg/kg), riboflavin (14 mg/kg), pantothenic acid (23 mg/kg), niacin (209 mg/kg), pyridoxine (27 mg/kg), folic acid (3 mg/kg), biotin (0.3 mg/kg) vitamin B_12_ (0.14 mg/kg). ^€^ Calculated from analyticals using modified Atwater numbers (kcal/g of 3.5 for protein, 8.5 for fat and 3.5 for nitrogen free extract). ^£^ Sum of the saturated fatty acids: 8:0 + 10:0 + 11:0 + 12:0 + 14:0 + 15:0 + 16:0 + 17:0 + 18:0 + 20:0 + 22:0 + 24:0. ^¥^ Sum of the monounsaturated fatty acids: 14:1 + 15:1 + 16:1 + 17:1 + 18:1 + 20:1 + 22:1 + 24:1. ^π^ Sum of the polyunsaturated fatty acids: 18:2(n-6) + 18:3(n-6) + 18:3(n-3) + 18:4(n-3) + 20:2(n-6) + 20:3(n-6) + 20:3(n-3) + 20:4(n-6) + 20:4(n-3) + 20:5(n-3) + 21:5(n-3) + 22:2(n-6) + 22:4(n-6) + 22:5(n-6) + 22:5(n-3) + 22:6(n-3). ^Ω^ Sum of the (n-6) fatty acids. ^θ^ Sum of the (n-3) fatty acids.

**Table 2:** Body weight and selected serum biochemistries from cats eating control food (COF), or test (E&DF) COF enhanced with EPA and DHA from fish oil, or test food 2 (ARAF) COF enhanced with arachidonic acid from chicken liver, at initial, 56 days, 84 days of test and change ± during the study (values are lsmeans ± standard errors).

Analyte	Control (COF Group)	E&DF Group	ARAF Group	F-test *p* Value
Body Weight (kg) Initial	5.21 ± 0.34	5.07 ± 0.33	4.84 ± 0.33	0.74
Body Weight (kg) 56 day	5.00 ± 0.30	4.89 ± 0.30	4.74 ± 0.29	0.83
Body Weight (kg) 84 day	4.94 ± 0.28	4.76 ± 0.28	4.64 ± 0.27	0.74
Body Weight (kg) Change	−0.27 ± 0.11	−0.31 ± 0.11	-0.20 ± 0.10	0.16
Albumin (mg/dl) Initial	3.67 ± 0.05	3.74 ± 0.05	3.79 ± 0.05	0.32
Albumin (mg/dl) 56 day	3.89 ± 0.05 ^a^	3.93 ± 0.05 ^a,b^	4.05 ± 0.05 ^b^	0.08
Albumin (mg/dl) 84 day	3.89 ± 0.06	3.92 ± 0.06	4.06 ± 0.06	0.15
Albumin (mg/dl) Change	0.22 ± 0.05	0.16 ± 0.05	0.27 ± 0.05	0.36
Total Protein (mg/dl) Initial	6.68 ± 0.09	6.73 ± 0.08	6.79 ± 0.09	0.67
Total Protein (mg/dl) 56 day	6.61 ± 0.11	6.81 ± 0.11	6.80 ± 0.11	0.33
Total Protein (mg/dl) 84 day	6.42 ± 0.10	6.55 ± 0.10	6.57 ± 0.09	0.47
Total Protein (mg/dl) Change	−0.26 ± 0.07	−0.22 ± 0.07	−0.23 ± 0.07	0.87
Urea Nitrogen (mg/dl) Initial	20.1 ± 0.8	19.2 ± 0.8	19.7 ± 0.8	0.67
Urea Nitrogen (mg/dl) 56 day	21.5 ± 0.9	19.8 ± 0.9	19.8 ± 0.8	0.32
Urea Nitrogen (mg/dl) 84 day	22.7 ± 1.0 ^a^	18.9 ± 1.0 ^b^	19.5 ± 0.9 ^b^	0.02
Urea Nitrogen (mg/dl) Change	2.5 ± 0.8 ^a^	0.1 ± 0.8u ^b^	−0.4 ± 0.8 ^b^	0.03
Creatinine (mg/dl) Initial	1.17 ± 0.05	1.11 ± 0.05	1.15 ± 0.05	0.80
Creatinine (mg/dl) 56 day	1.19 ± 0.05	1.20 ± 0.05	1.19 ± 0.05	0.98
Creatinine (mg/dl) 84 day	1.25 ± 0.05	1.13 ± 0.05	1.20 ± 0.05	0.40
Creatinine (mg/dl) Change	0.08 ± 0.03	0.01 ± 0.03	0.06 ± 0.03	0.28
Triglycerides (mg/dl) Initial	34.6 ± 3.1	35.5 ± 3.3	35.0 ± 3.3	0.98
Triglycerides (mg/dl) 56 day	42.4 ± 9.0	50.8 ± 9.0	43.6 ± 8.6	0.77
Triglycerides (mg/dl) 84 day	37.2 ± 14.1	59.5 ± 14.1	39.4 ± 13.5	0.47
Triglycerides (mg/dl) Change	2.5 ± 13.8	22.7 ± 13.8	4.3 ± 13.8	0.52
Cholesterol (mg/dl) Initial	145.5 ± 7.2	140.5 ± 6.9	144.3 ± 7.2	0.87
Cholesterol (mg/dl) 56 day	152.0 ± 8.5 ^a,b^	134.4 ± 8.5 ^a^	169.0 ± 8.1 ^b^	0.02
Cholesterol (mg/dl) 84 day	153.6 ± 8.7 ^a,b^	130.9 ± 8.7 ^a^	161.4 ± 8.4 ^b^	0.05
Cholesterol (mg/dl) Change	8.2 ± 5.5 ^a^	−9.1 ± 5.5 ^b^	19.9 ± 5.5 ^a^	<0.01

^a,b^ Means with different superscripts in the same line are different using post-hoc PDIFF in SAS (*p* ≤ 0.05).

**Table 3:** Circulating concentration (mg/dL) of polyunsaturated fatty from cats eating control food (COF), or test food 1 (E&DF) COF enhanced with EPA and DHA from fish oil, or test food 2 (ARAF) COF enhanced with arachidonic acid from chicken liver, at initial, 56 days, 84 days of test and change during the study (values are lsmeans ± standard errors).

Analyte	Control	E&D Food Group	ARA Food Group	F-test *p* Value
LA [18:2 (n-6)] Initial	36.0 ± 2.1	35.5 ± 2.0	38.2 ± 2.1	0.62
LA [18:2 (n-6)] 56 day	45.8 ± 3.5 ^b^	33.5 ± 3.5 ^a^	46.1 ± 3.4 ^b^	0.02
LA [18:2 (n-6)] 84 day	40.8 ± 2.4 ^b^	30.3 ± 2.4 ^a^	38.7 ± 2.4 ^b^	0.01
LA [18:2 (n-6)] Change	4.8 ± 1.8 ^b^	−5.4 ± 1.8 ^a^	0.5± 1.8 ^b^	<0.01
αLA [18:3 (n-3)] Initial	1.1 ± O.1	1.1 ± O.1	1.1 ± O.1	0.87
αLA [18:3 (n-3)] 56 day	1.3 ± O.1	1.0 ± O.1	1.1 ± O.1	0.14
αLA [18:3 (n-3)] 84 day	1.3 ± O.1	1.1 ± O.1	1.1 ± O.1	0.16
αLA [18:3 (n-3)] Change	0.2 ± O.1	−0.1 ± O.1	0.0 ± O.1	0.12
ARA [20:4 (n-6)] Initial	20.0 ± 1.0	21.1 ± 0.9	22.0 ± 1.0	0.32
ARA [20:4 (n-6)] 56 day	20.8 ± 1.5 ^a^	18.3 ± 1.5 ^a^	31.4 ± 1.5 ^b^	<0.01
ARA [20:4 (n-6)] 84 day	21.5 ± 1.6 ^a^	17.1 ± 1.5 ^a^	31.1 ± 1.5 ^b^	<0.01
ARA [20:4 (n-6)] Change	1.6 ± 1.3 ^a^	−3.8 ± 1.3 ^b^	9.6 ± 1.3 ^c^	<0.01
EPA [20:5 (n-3)] Initial	0.5 ± 0.03	0.5 ± 0.03	0.5 ± 0.03	0.17
EPA [20:5 (n-3)] 56 day	0.5 ± 0.5 ^a^	5.7 ± 0.5 ^b^	0.6 ± 0.5 ^a^	<0.01
EPA [20:5 (n-3)] 84 day	0.6 ± 0.4 ^a^	5.4 ± 0.4 ^b^	0.6 ± 0.4 ^a^	<0.01
EPA [20:5 (n-3)] Change	0.1 ± 0.4 ^a^	4.9 ± 0.4 ^b^	0.1 ± 0.4 ^a^	<0.01
DHA [22:6 (n-3)] Initial	1.7 ± 0.1	1.8 ± 0.1	1.8 ± 0.1	0.72
DHA [22:6 (n-3)] 56 day	1.9 ± 0.4 ^a^	6.1 ± 0.4 ^b^	2.6 ± 0.4 ^a^	<0.01
DHA [22:6 (n-3)] 84 day	1.7 ± 0.4 ^a^	6.0 ± 0.4 ^b^	2.5 ± 0.4 ^a^	<0.01
DHA [22:6 (n-3)] Change	0.0 ± 0.4 ^a^	4.2 ± 0.4 ^b^	0.8 ± 0.4 ^a^	<0.01
ARA/(EPA+DHA) Initial	9.2 ± 0.3	9.0 ± 0.3	9.4 ± 0.3	0.52
ARA/(EPA+DHA) 56 day	8.8 ± 0.3 ^a^	1.7 ± 0.3 ^b^	9.7 ± 0.3 ^a^	<0.01
ARA/(EPA+DHA) 84 day	9.4 ± 0.3 ^a^	1.7 ± 0.3 ^b^	10.0 ± 0.3 ^a^	<0.01
ARA/(EPA+DHA) Change	0.2 ± 0.4 ^a^	−7.3 ± 0.4 ^b^	0.6 ± 0.4 ^a^	<0.01
Sum of n-3 ^£^ Initial	4.4 ± 0.29	4.7 ± 0.2	4.6 ± 0.2	0.48
Sum of n-3 ^£^ 56 day	4.9 ± 0.8 ^b^	14.7 ± 0.8 ^a^	5.9 ± 0.7 ^b^	<0.01
Sum of n-3 ^£^ 84 day	4.7 ± 0.8 ^b^	14.2 ± 0.8 ^a^	5.1 ± 0.8 ^b^	<0.01
Sum of n-3 ^£^ Change	0.3 ± 0.8 ^b^	9.5 ± 0.8 ^a^	0.9 ± 0.8 ^b^	<0.01
Sum of n-6 ^£^ Initial	61.0 ± 2.7	61.6 ± 2.6	65.4 ± 2.7	0.45
Sum of n-6 ^θ^ 56 day	71.8 ± 4.8 ^a^	55.0 ± 4.8 ^b^	82.6 ± 4.6 ^a^	<0.01
Sum of n-6 ^θ^ 84 day	68.0 ± 4.0 ^a^	50.7 ± 4.0 ^b^	75.0 ± 3.8 ^a^	<0.01
Sum of n-6 ^θ^ Change	7.0 ± 2.8 ^a^	−10.8 ± 2.8 ^b^	10.9 ± 2.8 ^a^	<0.01
Sum of PUFA ^¥^ Initial	65.3 ± 2.8	66.3 ± 2.7	70.0 ± 2.8	0.46
Sum of PUFA ^¥^ 56 day	76.7 ± 5.1 ^a,b^	69.7 ± 5.1 ^a^	88.3 ± 4.9 ^b^	0.04
Sum of PUFA^¥^ 84 day	72.7 ± 4.3 ^a,b^	64.9 ± 4.3 ^a^	80.4 ± 4.1 ^b^	0.05
Sum of PUFA ^¥^ Change	7.4 ± 2.8 ^b^	−1.4 ± 2.8 ^a^	11.8 ± 2.8 ^b^	<0.01
(n-6):(n-3) ratio Initial	13.9 ± 0.4	13.3 ± 0.4	14.1 ± 0.4	0.32
(n-6):(n-3) ratio 56 day	14.8 ± 0.5 ^a^	4.0 ± 0.5 ^b^	14.4 ± 0.5 ^a^	<0.01
(n-6):(n-3) ratio 84 day	14.4 ± 0.4 ^a^	3.9 ± 0.4 ^b^	13.8 ± 0.4 ^a^	<0.01
(n-6):(n-3) ratio Change	0.4 ± 0.5 ^a^	−9.5 ± 0.5 ^b^	−0.3 ± 0.5 ^a^	<0.01

^£^ Sum of all n-3 fatty acids defined below. ^θ^ Sum of all n-6 fatty acids defined below. ^¥^ Sum of the polyunsaturated fatty acids: 18:2(n-6) + 18:3(n-6) + 18:3(n-3) + 18:4(n-3) + 20:2(n-6) + 20:3(n-6) + 20:3(n-3) + 20:4(n-6) + 20:4(n-3) + 20:5(n-3) + 21:5(n-3) + 22:2(n-6) + 22:4(n-6) + 22:5(n-6) + 22:5(n-3) + 22:6(n-3). ^a,b,c^ Means with different superscripts in the same line are different (*p* ≤ 0.05).

**Table 4:** Circulating concentration (mg/dL) of saturated and monounsaturated fatty acids from cats eating control food (COF), or test food 1 (E&DF) COF enhanced with EPA and DHA from fish oil, or test food 2 (ARAF) COF enhanced with arachidonic acid from chicken liver, at initial, 56 days, 84 days of test and change during the study (values are lsmeans ± standard errors).

Analyte	Control	E&D Food Group	ARA Food Group	F-test *p* Value
Myristic acid [14:0] Initial	0.51 ± 0.13	0.83 ± 0.12	0.60 ± 0.13	0.17
Myristic acid [14:0] 56 day	0.55 ± 0.08	0.44 ± 0.08	0.42 ± 0.07	0.46
Myristic acid [14:0] 84 day	0.48 ± 0.13	0.53 ± 0.13	0.64 ± 0.12	0.67
Myristic acid [14:0] Change	−0.02 ± 0.17	−0.27 ± 0.17	0.26 ± 0.17	0.45
Palmitic acid [16:0] Initial	19.7 ± 1.5	23.2 ± 1.5	22.3 ± 1.6	0.28
Palmitic acid [16:0] 56 day	23.6 ± 1.7 ^a,b^	20.7 ± 1.6 ^a^	25.6 ± 1.5 ^b^	0.09
Palmitic acid [16:0] 84 day	22.3 ± 1.8 ^a,b^	20.9 ± 1.8 ^a^	26.1 ± 1.7 ^b^	0.11
Palmitic acid [16:0] Change	2.6 ± 2.0 ^a,b^	−1.9 ± 2.0 ^a^	4.0 ± 2.0 ^b^	0.11
Stearic acid [18:0] Initial	38.4 ± 2.1	41.7 ± 2.0	43.2 ± 2.1	0.25
Stearic acid [18:0] 56 day	42.5 ± 2.8 ^a,b^	40.1 ± 2.8 ^a^	50.3 ± 2.7 ^b^	0.04
Stearic acid [18:0] 84 day	43.5 ± 3.1 ^a,b^	39.8 ± 3.1 ^a^	51.7 ± 2.9 ^b^	0.02
Stearic acid [18:0] Change	5.1 ± 2.5 ^a,b^	−1.5 ± 2.5 ^a^	9.3 ± 2.5 ^b^	0.01
Palmitoleic acid [16:1] Initial	1.5 ± 0.14	1.6 ± 0.13	1.4 ± 0.14	0.71
Palmitoleic acid [16:1] 56 day	1.6 ± 0.09 ^a^	1.1 ± 0.09 ^b^	1.2 ± 0.09 ^b^	0.01
Palmitoleic acid [16:1] 84 day	1.5 ± 0.11 ^a^	1.1 ± 0.11 ^b^	1.3 ± 0.10 ^a,b^	0.08
Palmitoleic acid [16:1] Change	0.01 ± 0.14 ^a^	−0.4 ± 0.14 ^b^	−0.1 ± 0.14 ^a,b^	0.06
Oleic acid [18:1] Initial	22.2 ± 1.8	23.7 ± 1.7	22.5 ± 1.8	0.81
Oleic acid [18:1] 56 day	25.8 ± 1.6 ^a^	18.9 ± 1.6 ^b^	22.9 ± 1.6 ^a,b^	0.05
Oleic acid [18:1] 84 day	26.5 ± 1.8 ^a^	20.2 ± 1.8 ^b^	25.1 ± 1.8 ^a,b^	0.05
Oleic acid [18:1] Change	4.3 ± 1.8 ^a^	−3.4 ± 1.8 ^b^	2.7 ± 1.8 ^a^	0.01
SFA ^£^ Initial	58.6 ± 3.5	65.7 ± 3.4	66.1 ± 3.5	0.25
SFA ^£^ 56 day	66.7 ± 4.3 ^a,b^	61.3 ± 4.3 ^a^	76.3 ± 4.1 ^b^	0.05
SFA ^£^ 84 day	66.4 ± 4.7 ^a,b^	61.3 ± 4.7 ^a^	78.4 ± 4.5 ^b^	0.03
SFA ^£^ Change	7.8 ± 4.4 ^a,b^	−3.6 ± 4.4 ^a^	13.4 ± 4.4 ^b^	0.03
MUFA^¥^ Initial	23.7 ± 1.9	25.3 ± 1.8	23.9 ± 1.9	0.81
MUFA ^¥^ 56 day	27.4 ± 1.6 ^a^	20.0 ± 1.6 ^b^	24.1 ± 1.6 ^a,b^	0.02
MUFA ^¥^ 84 day	28.0 ± 1.8 ^a^	21.4 ± 1.8 ^b^	26.4 ± 1.8 ^a,b^	0.05
MUFA ^¥^ Change	4.3 ± 2.0 ^a^	−3.8 ± 2.0 ^b^	2.6 ± 2.0 ^a^	0.01

^£^ Sum of the saturated fatty acids: 8:0 + 10:0 + 11:0 + 12:0 + 14:0 + 15:0 + 16:0 + 17:0 + 18:0 + 20:0 + 22:0 + 24:0. ^¥^ Sum of the monounsaturated fatty acids: 14:1 + 15:1 + 16:1 + 17:1 + 18:1 + 20:1 + 22:1 + 24:1. ^a,b^ Means with different superscripts in the same line are different (*p* ≤ 0.05).

**Table 5:** Change in or end of study difference in selected analytes from plasma metabolomics screening of cats eating control food (COF), or test food 1 (E&DF) COF enhanced with EPA and DHA from fish oil, or test food 2 (ARAF) COF enhanced with arachidonic acid from chicken liver.*

Biochemical	Change in Control Food (COF) Group	Change in E&D Food (E&DF) Group	Change in ARA Food (ARAF) Group	E&DF to COF Group End of Study	ARAF to COF Groups End of Study	ARAF to E&DF Groups End of Study
**Amino acid metabolites**						
Sarcosine	0.53	0.37	0.4	0.69	0.75	1.09
Dimethylglycine	1.03	0.95	0.88	0.81	0.78	0.96
Betaine	1.44	0.89	1.02	0.56	0.69	1.23
1-methyl-4-imidazoleacetate	0.85	0.76	1.16	0.73	1.32	1.82
1-ribosyl-imidazoleacetate	0.55	0.52	0.65	0.77	1.39	1.8
4-imidazoleacetate	0.62	0.61	0.88	0.65	1.2	1.84
N-acetylhistamine	1	1.34	2.65	0.64	2.32	3.66
Urea	1.15	1.01	0.98	0.83	0.85	1.04
N-delta-acetylornithine	0.38	0.35	0.4	0.67	0.99	1.47
5-oxoproline	1.07	1.09	1.02	1.16	1.01	0.87
**Postbiotics**						
phenol sulfate	1.11	0.78	1.48	0.51	1.24	2.42
4-methoxyphenol sulfate	3.89	2.4	6.03	0.27	0.96	3.52
2-hydroxyphenylacetate	1.04	0.87	1.51	0.81	1.28	1.58
Indoleacetate	0.91	1.13	1.49	0.64	1.33	2.08
Indolepropionate	1.03	0.88	1.38	0.69	1.31	1.9
Indoleacetylglutamine	1.1	1.04	1.7	0.55	1.3	2.37
Phenylacetylglutamate	1.63	1.14	1.58	0.62	0.87	1.41
Phenylacetylglutamine	1.73	1.14	1.62	0.51	0.87	1.72
Phenylacetylglycine	1.45	1.16	1.16	0.59	0.71	1.21
Phenylacetylserine	1.87	1.27	1.24	0.29	0.43	1.49
3-methyl catechol sulfate	1.75	0.89	2.44	0.18	0.62	3.51
4-methylcatechol sulfate	1.16	1.44	2.01	0.60	0.88	1.47
4-ethylphenylsulfate	1.6	0.7	1.51	0.35	0.71	2.00
4-vinylphenol sulfate	1.16	0.6	1.02	0.34	0.65	1.93
p-cresol sulfate	1.34	1.23	1.4	0.50	0.75	1.49
**Lipids**						
myristate (14:0)	1.03	1.45	1.14	1.33	1.01	0.75
heneicosapentaenoate (21:5n3)	1	20.07	1.05	20.00	1.05	0.05
hexadecadienoate (16:2n6)	0.86	1.83	0.9	2.22	1.03	0.46
hexadecatrienoate (16:3n3)	1.63	13.64	1.47	14.29	1.03	0.07
stearidonate (18:4n3)	0.81	21.87	1.2	25.10	1.3	0.05
eicosapentaenoate (EPA; 20:5n3)	0.89	24.68	1.23	32.26	1.36	0.04
docosapentaenoate (n3 DPA; 22:5n3)	0.86	3.19	1.1	4.35	1.29	0.29
docosahexaenoate (DHA; 22:6n3)	0.77	6.26	1.41	9.09	1.73	0.19
arachidonate (20:4n6)	1.01	1.02	1.26	1.22	1.52	1.25
adrenate (22:4n6)	0.99	0.9	1.41	1.14	1.31	1.15
docosapentaenoate (n6 DPA; 22:5n6)	0.91	1.31	1.9	1.61	2.15	1.33
sebacate (C10-DC)	1.11	2.06	1.37	1.37	1.17	0.86
myristoylcarnitine (C14)	1.29	1.57	1.38	1.54	1.14	0.75
arachidonoylcarnitine (C20:4)	1.26	1.06	1.74	0.97	1.64	1.69
adrenoylcarnitine (C22:4)	1.33	0.89	1.73	0.78	1.58	2.01
cerotoylcarnitine (C26)	1.28	1.31	1.51	1.39	1.38	0.99
3-hydroxybutyrate (BHBA)	1.07	1.29	1.69	1.61	1.73	1.08
1-palmitoyl-2-oleoyl-GPC (16:0/18:1)	1.08	0.98	1.06	0.90	1.01	1.11
1-palmitoyl-2-linoleoyl-GPC (16:0/18:2)	1.04	0.85	1.03	0.83	1.07	1.28
1-palmitoyl-2-arachidonoyl-GPC (16:0/20:4n6)	1.03	0.88	1.23	0.89	1.35	1.51
1-palmitoyl-2-docosahexaenoyl-GPC (16:0/22:6)	1.08	3.76	1.59	3.70	1.71	0.46
1-palmitoleoyl-2-linoleoyl-GPC (16:1/18:2)	1.13	0.58	0.92	0.52	0.83	1.58
1-palmitoleoyl-2-linolenoyl-GPC (16:1/18:3) *	1.3	0.54	0.92	0.45	0.72	1.62
1,2-distearoyl-GPC (18:0/18:0)	1.13	0.7	1	0.60	0.8	1.35
1-stearoyl-2-oleoyl-GPC (18:0/18:1)	1.09	0.96	1.04	0.85	0.96	1.14
1-stearoyl-2-linoleoyl-GPC (18:0/18:2)	1.03	0.91	0.99	0.87	1.02	1.18
1-stearoyl-2-arachidonoyl-GPC (18:0/20:4)	1	0.95	1.21	0.95	1.28	1.35
1-stearoyl-2-docosahexaenoyl-GPC (18:0/22:6)	1.13	4.13	1.65	3.70	1.71	0.46
1-oleoyl-2-docosahexaenoyl-GPC (18:1/22:6) *	1.24	2.38	1.21	1.92	0.89	0.47
1,2-dilinoleoyl-GPC (18:2/18:2)	1.1	0.56	0.86	0.51	0.78	1.54
1-linoleoyl-2-linolenoyl-GPC (18:2/18:3)	1.52	0.79	1.03	0.56	0.76	1.36
1-linoleoyl-2-arachidonoyl-GPC (18:2/20:4n6) *	1.04	0.84	1.03	0.78	1.01	1.29
1-palmitoyl-2-linoleoyl-GPE (16:0/18:2)	1.33	1.33	0.83	1.05	0.82	0.79
1-palmitoyl-2-arachidonoyl-GPE (16:0/20:4) *	1.21	0.86	0.96	0.78	1.12	1.44
1-palmitoyl-2-docosahexaenoyl-GPE (16:0/22:6) *	1.21	6.41	1.8	5.56	2.2	0.4
1-stearoyl-2-linoleoyl-GPE (18:0/18:2)	1.27	1.09	0.92	0.81	0.88	1.09
1-stearoyl-2-arachidonoyl-GPE (18:0/20:4)	1.08	1.08	1.19	0.94	1.43	1.51
1-oleoyl-2-linoleoyl-GPE (18:1/18:2) *	1.46	0.79	0.81	0.51	0.6	1.18
1-palmitoyl-2-oleoyl-GPI (16:0/18:1)	1.24	0.94	1.12	0.67	0.84	1.25
1-palmitoyl-2-linoleoyl-GPI (16:0/18:2)	1.3	1.09	1.09	0.74	0.88	1.2
1-palmitoyl-2-arachidonoyl-GPI (16:0/20:4) *	1.09	1	1.22	0.85	1.29	1.5
1-stearoyl-2-oleoyl-GPI (18:0/18:1)	1.2	0.93	1.08	0.70	0.75	1.06
1-stearoyl-2-linoleoyl-GPI (18:0/18:2)	1.37	0.96	1.06	0.72	0.83	1.15
1-oleoyl-2-linoleoyl-GPI (18:1/18:2)	1.47	0.88	1.09	0.57	0.74	1.31
1-stearoyl-2-arachidonoyl-GPI (18:0/20:4)	1.09	0.96	1.12	0.88	1.12	1.27
1-palmitoleoyl-GPC (16:1) *	1.04	0.65	0.8	0.65	0.74	1.15
1-oleoyl-GPC (18:1)	1.1	0.82	0.95	0.72	0.81	1.12
1-linoleoyl-GPC (18:2)	0.98	0.72	0.89	0.72	0.88	1.21
1-linolenoyl-GPC (18:3)	1.07	0.75	0.98	0.75	0.91	1.21
1-arachidonoyl-GPC (20:4n6)	1.02	0.76	1.31	0.74	1.36	1.83
1-lignoceroyl-GPC (24:0)	1.16	0.96	1.1	0.86	1.13	1.32
1-palmitoyl-GPE (16:0)	1.09	2.03	1.1	1.89	1.23	0.65
1-oleoyl-GPE (18:1)	1.36	0.72	1.08	0.51	0.68	1.36
1-linoleoyl-GPE (18:2)	1.2	0.6	0.95	0.53	0.83	1.55
1-arachidonoyl-GPE (20:4n6)	1.13	0.64	1.27	0.66	1.28	1.95
1-stearoyl-GPG (18:0)	1.29	0.84	1.06	0.68	1.05	1.56
1-linoleoyl-GPG (18:2)	1.17	0.66	1.09	0.53	0.96	1.83
1-oleoyl-GPI (18:1)	1.58	1.14	1.51	0.57	0.75	1.34
1-(1-enyl-palmitoyl)-2-oleoyl-GPE (P-16:0/18:1) *	0.76	0.51	0.67	0.50	0.7	1.39
1-(1-enyl-palmitoyl)-2-linoleoyl-GPE (P-16:0/18:2)	0.58	0.38	0.52	0.46	0.67	1.45
1-(1-enyl-palmitoyl)-2-palmitoleoyl-GPC (P-16:0/16:1)	0.85	0.46	0.75	0.46	0.75	1.64
1-(1-enyl-palmitoyl)-2-arachidonoyl-GPE (P-16:0/20:4)	0.91	0.67	1.08	0.67	1.16	1.72
1-(1-enyl-stearoyl)-2-oleoyl-GPE (P-18:0/18:1)	0.8	0.53	0.76	0.54	0.88	1.62
1-(1-enyl-stearoyl)-2-linoleoyl-GPE (P-18:0/18:2)	0.77	0.51	0.7	0.58	0.9	1.55
1-(1-enyl-palmitoyl)-2-arachidonoyl-GPC (P-16:0/20:4)	0.89	0.65	1.29	0.74	1.48	2.02
1-(1-enyl-palmitoyl)-2-linoleoyl-GPC (P-16:0/18:2)	1.04	0.53	0.87	0.47	0.8	1.68
1-(1-enyl-stearoyl)-2-arachidonoyl-GPE (P-18:0/20:4)	0.94	0.67	1.21	0.73	1.46	2
palmitoleoyl-linoleoyl-glycerol (16:1/18:2)	1.18	0.68	1.12	0.55	0.9	1.63
stearoyl-arachidonoyl-glycerol (18:0/20:4)	1.29	0.86	1.32	0.71	1.16	1.63
stearoyl-arachidonoyl-glycerol (18:0/20:4)	1.21	0.93	1.43	0.78	1.29	1.66
oleoyl-arachidonoyl-glycerol (18:1/20:4)	1.02	0.72	1.46	0.74	1.63	2.2
oleoyl-arachidonoyl-glycerol (18:1/20:4)	1.14	0.88	1.62	0.81	1.58	1.95
linoleoyl-arachidonoyl-glycerol (18:2/20:4)	1.1	1.97	1.86	2.00	2.09	1.04
Sphinganine	0.93	0.74	1	0.8	1.03	1.3
sphinganine-1-phosphate	1	0.65	0.86	0.67	1.04	1.54
palmitoyl sphingomyelin (d18:1/16:0)	1.04	0.85	1.08	0.81	1	1.24
stearoyl sphingomyelin (d18:1/18:0)	1.02	1.15	1.17	1.14	1.15	1.01
behenoyl sphingomyelin (d18:1/22:0)	1.08	0.98	1.18	0.85	0.99	1.17
tricosanoyl sphingomyelin (d18:1/23:0)	1.24	1.01	1.25	0.80	0.96	1.2
lignoceroyl sphingomyelin (d18:1/24:0)	1.26	1.06	1.19	0.83	0.92	1.11
sphingomyelin (d18:2/18:1)	1.09	0.78	1	0.71	0.86	1.2
sphingomyelin (d17:1/14:0, d16:1/15:0)	1.18	0.89	1.05	0.66	0.71	1.08
sphingomyelin (d18:1/14:0, d16:1/16:0)	1.05	0.88	0.96	0.73	0.74	0.98
sphingomyelin (d18:2/14:0, d18:1/14:1)	1.28	0.95	1.03	0.67	0.67	1
sphingomyelin (d17:1/16:0, d18:1/15:0, d16:1/17:0)	1.04	0.84	1.08	0.81	0.93	1.15
sphingomyelin (d17:2/16:0, d18:2/15:0)	1.27	1.04	1.27	0.76	0.91	1.19
sphingomyelin (d18:2/16:0, d18:1/16:1)	1.08	0.85	1.06	0.75	0.95	1.26
sphingomyelin (d18:1/18:1, d18:2/18:0)	1.08	0.9	1.11	0.87	1.08	1.26
sphingomyelin (d18:1/21:0, d17:1/22:0, d16:1/23:0)	1.1	0.87	1.05	0.76	0.82	1.08
Cholesterol	1.03	0.91	1.15	0.81	1.12	1.38
7-alpha-hydroxy-3-oxo-4-cholestenoate (7-Hoca)	0.94	0.84	0.79	0.76	1.1	1.45
3beta-hydroxy-5-cholestenoate	0.92	0.59	0.69	0.52	1.06	2.05
4-cholesten-3-one	1.24	1.05	1.51	0.85	1.22	1.43
Campesterol	1.13	0.97	1.3	0.78	1.07	1.37
**Nucleotides, vitamins**						
Guanine	0.67	0.79	0.53	1.41	0.89	0.63
5-hydroxymethylcytidine	1.75	1.75	1.22	1.08	0.49	0.45
N1-Methyl-2-pyridone-5-carboxamide	1.21	1.01	1.16	0.53	1.01	1.88
alpha-tocopherol	1.2	1.13	1.33	0.89	1.08	1.21
Pyridoxate	2.06	1.79	1.68	0.8	0.69	0.87
2-isopropylmalate	0.66	0.83	1	1.18	2.34	1.98
equol sulfate	1.22	1.35	1.79	0.84	1.72	2.04
stachydrine	0.75	0.56	0.58	0.69	0.77	1.12
4-vinylguaiacol sulfate	1.22	0.87	1.12	0.66	0.79	1.42
2,5-dimethylphenol sulfate	1.24	1.24	1.45	0.87	1.1	1.26
2,4-dichlorophenol sulfate	1.64	1.27	1.85	0.75	1.55	2.06

* This table consists of all analytes that had a *p* ≤ 0.05 and a *q* ≤ 0.1 for both change during the study feeding period (green is decreased while red connotes increase) and a difference between treatments at the end of the study. Green denotes a decline and red an increase in the comparison of that column. Values are the means of the ratios of the initial values divided by final values (change within groups) or ratios of the mean values at the end of the study (change between groups).

## References

[1] Schoenherr W., Jewell D.E. (1997). Nutritional modification of inflammatory diseases. Semin. Vet. Med. Surg. Small Anim..

[2] Park H.J., Park J.S., Hayek M.G., Reinhart G.A., Chew B.P. (2011). Dietary fish oil and flaxseed oil suppress inflammation and immunity in cats. Vet. Immunol. Immunopathol..

[3] Wander R., Hall J., Gradin J.L., Du S.-H., Jewell D.E. (1997). The ratio of dietary (n-6) to (n-3) fatty acids influences immune system function, eicosanoid metabolism, lipid peroxidation and vitamin E status in aged dogs. J. Nutr..

[4] Świątkiewicz S., Arczewska-Wlosek A., Józefiak D. (2015). The relationship between dietary fat sources and immune response in poultry and pigs: An updated review. Livest. Sci..

[5] Galli C., Calder P.C. (2009). Effects of Fat and Fatty Acid Intake on Inflammatory and Immune Responses: A Critical Review. Ann. Nutr. Metab..

[6] Jackson M.I., Jewell D.E. (2020). Docosahexaenoate-enriched fish oil and medium chain triglycerides shape the feline plasma lipidome and synergistically decrease circulating gut microbiome-derived putrefactive postbiotics. PLoS ONE.

[7] Wikoff W.R., Anfora A.T., Liu J., Schultz P.G., Lesley S.A., Peters E.C., Siuzdak G. (2009). Metabolomics analysis reveals large effects of gut microflora on mammalian blood metabolites. Proc. Natl. Acad. Sci. USA.

[8] Dodd D., Spitzer M.H., Van Treuren W., Merrill B.D., Hryckowian A.J., Higginbottom S.K., Le A., Cowan T.M., Nolan G.P., Fischbach M.A. (2017). A gut bacterial pathway metabolizes aromatic amino acids into nine circulating metabolites. Nat. Cell Biol..

[9] Cui C., Li Y., Gao H., Zhang H., Han J., Zhang D., Li Y., Zhou J., Lu C., Su X. (2017). Modulation of the gut microbiota by the mixture of fish oil and krill oil in high-fat diet-induced obesity mice. PLoS ONE.

[10] Costantini L., Molinari R., Farinon B., Merendino N. (2017). Impact of Omega-3 Fatty Acids on the Gut Microbiota. Int. J. Mol. Sci..

[11] Diether N.E., Willing B.P. (2019). Microbial Fermentation of Dietary Protein: An Important Factor in Diet–Microbe–Host Interaction. Microorganisms.

[12] Mair R.D., Sirich T.L., Plummer N.S., Meyer T.W. (2018). Characteristics of Colon-Derived Uremic Solutes. Clin. J. Am. Soc. Nephrol..

[13] Poesen R., Claes K., Evenepoel P., De Loor H., Augustijns P., Kuypers D., Meijers B. (2016). Microbiota-Derived Phenylacetylglutamine Associates with Overall Mortality and Cardiovascular Disease in Patients with CKD. J. Am. Soc. Nephrol..

[14] Yang C.-Y., Tarng D.-C. (2018). Diet, gut microbiome and indoxyl sulphate in chronic kidney disease patients. Nephrology.

[15] Nemet I., Saha P.P., Gupta N., Zhu W., Romano K.A., Skye S.M., Cajka T., Mohan M.L., Li L., Wu Y. (2020). A Cardiovascular Disease-Linked Gut Microbial Metabolite Acts via Adrenergic Receptors. Cell.

[16] Holmes E., Li J.V., Athanasiou T., Ashrafian H., Nicholson J.K. (2011). Understanding the role of gut microbiome–host metabolic signal disruption in health and disease. Trends Microbiol..

[17] Parthasarathy A., Cross P.J., Dobson R.C.J., Adams L.E., Savka M.A., Hudson A.O. (2018). A Three-Ring Circus: Metabolism of the Three Proteogenic Aromatic Amino Acids and Their Role in the Health of Plants and Animals. Front. Mol. Biosci..

[18] Verbrugghe A., Bakovic M. (2013). Peculiarities of One-Carbon Metabolism in the Strict Carnivorous Cat and the Role in Feline Hepatic Lipidosis. Nutrients.

[19] Zhao G., He F., Wu C., Li P., Li N., Deng J., Zhu G., Ren W.-K., Peng Y. (2018). Betaine in Inflammation: Mechanistic Aspects and Applications. Front. Immunol..

[20] Wu J., He C., Bu J., Luo Y., Yang S., Ye C., Yu S., He B., Yin Y., Yang X. (2020). Betaine attenuates LPS-induced downregulation of Occludin and Claudin-1 and restores intestinal barrier function. BMC Vet. Res..

[21] García-Ródenas C.L., E Bergonzelli G., Nutten S., Schumann A., Cherbut C., Turini M., Ornstein K., Rochat F., Corthésy-Theulaz I. (2006). Nutritional Approach to Restore Impaired Intestinal Barrier Function and Growth After Neonatal Stress in Rats. J. Pediatr. Gastroenterol. Nutr..

[22] Zhang Y.-G., Xia Y., Lu R., Sun J. (2018). Inflammation and intestinal leakiness in older HIV+ individuals with fish oil treatment. Genes Dis..

[23] Abulizi N., Quin C., Brown K., Chan Y.K., Gill S.K., Gibson D.L. (2019). Gut Mucosal Proteins and Bacteriome Are Shaped by the Saturation Index of Dietary Lipids. Nutrients.

[24] Belkaid Y., Hand T.W. (2014). Role of the Microbiota in Immunity and Inflammation. Cell.

[25] Du J., Shen L., Tan Z., Zhang P., Zhao X., Xu Y., Gan M., Yang Q., Ma J., Jiang A. (2018). Betaine Supplementation Enhances Lipid Metabolism and Improves Insulin Resistance in Mice Fed a High-Fat Diet. Nutrients.

[26] Rizzo G., Laganà A.S. (2020). The Link between Homocysteine and Omega-3 Polyunsaturated Fatty Acid: Critical Appraisal and Future Directions. Biomolecules.

[27] Floerchinger A.M., Jackson M.I., Jewell D.E., MacLeay J.M., Paetau-Robinson I., Hahn K.A. (2015). Effect of feeding a weight loss food beyond a caloric restriction period on body composition and resistance to weight gain in dogs. J. Am. Vet. Med. Assoc..

[28] Hall J., Jewell D.E. (2012). Feeding Healthy Beagles Medium-Chain Triglycerides, Fish Oil, and Carnitine Offsets Age-Related Changes in Serum Fatty Acids and Carnitine Metabolites. PLoS ONE.

[29] Bright J.M., Sullivan P., Melton S.L., Schneider J.F., McDonald T.P. (1994). The Effects of n-3 Fatty Acid Supplementation on Bleeding Time, Plasma Fatty Acid Composition, and In Vitro Platelet Aggregation in Cats. J. Vet. Intern. Med..

[30] Hall J., Brockman J.A., Davidson S.J., MacLeay J.M., Jewell D.E. (2017). Increased dietary long-chain polyunsaturated fatty acids alter serum fatty acid concentrations and lower risk of urine stone formation in cats. PLoS ONE.

[31] Balk E.M., Lichtenstein A.H., Chung M., Kupelnick B., Chew P., Lau J. (2006). Effects of omega-3 fatty acids on serum markers of cardiovascular disease risk: A systematic review. Atheroscler..

[32] Bernstein A.M., Ding E.L., Willett W.C., Rimm E.B. (2012). A meta-analysis shows that docosahexaenoic acid from algal oil reduces serum triglycerides and increases HDL-cholesterol and LDL-cholesterol in persons without coronary heart disease. J. Nutr..

[33] Hess H.A., Corl B.A., Lin X., Jacobi S.K., Harrell R.J., Blikslager A., Odle J. (2008). Enrichment of Intestinal Mucosal Phospholipids with Arachidonic and Eicosapentaenoic Acids Fed to Suckling Piglets Is Dose and Time Dependent. J. Nutr..

[34] Ye J., Lv L., Wu W., Li Y., Shi D., Fang D., Guo F., Jiang H., Yan R., Ye W. (2018). Butyrate Protects Mice Against Methionine–Choline-Deficient Diet-Induced Non-alcoholic Steatohepatitis by Improving Gut Barrier Function, Attenuating Inflammation and Reducing Endotoxin Levels. Front. Microbiol..

[35] Zhuang P., Shou Q., Lu Y., Wang G., Qiu J., Wang J., He L., Chen J., Jiao J., Zhang Y. (2017). Arachidonic acid sex-dependently affects obesity through linking gut microbiota-driven inflammation to hypothalamus-adipose-liver axis. Biochim. Biophys. Acta (BBA)—Mol. Basis Dis..

[36] Ramakers J.D., Mensink R.P., Schaart G., Plat J. (2007). Arachidonic Acid but not Eicosapentaenoic Acid (EPA) and Oleic Acid Activates NF-κB and Elevates ICAM-1 Expression in Caco-2 Cells. Lipids.

[37] Mödinger Y., Schön C., Wilhelm M., Hals P.-A. (2019). Plasma Kinetics of Choline and Choline Metabolites after A Single Dose of SuperbaBoostTM Krill Oil or Choline Bitartrate in Healthy Volunteers. Nutrients.

[38] Piolot A., Blache D., Boulet L., Fortin L.J., Dubreuil D., Marcoux C., Davignon J., Lussier-Cacan S. (2003). Effect of fish oil on LDL oxidation and plasma homocysteine concentrations in health. J. Lab. Clin. Med..

[39] Dong X., Wang J., Ji P., Gao X., Sun L., Miao S., Lei Y., Du X., Zhang X. (2020). Dietary betaine supplementation promotes growth, n-3 LC-PUFA content and innate immunity in Macrobrachium rosenbergii. Aquaculture..

[40] Dong L., Zhong Z.X., Cui H.H., Wang S.N., Luo Y., Yu L.H., Loor J.J., Wang H. (2020). Effects of rumen-protected betaine supplementation on meat quality and the composition of fatty and amino acids in growing lambs. Animals.

[41] Kaur G. (2019). Parenteral Betaine as a Strategy to Prevent Fatty Liver and Improve Docosahexaenoic Acid and Arachidonic Acid Distribution in Parenterally Fed Neonatal Piglets. Master’s Thesis.

[42] Goldstein L., Davis E.M. (1994). Taurine, betaine, and inositol share a volume-sensitive transporter in skate erythrocyte cell membrane. Am. J. Physiol. Integr. Comp. Physiol..

[43] Varatharajalu R., Garige M., Leckey L.C., Gong M., Lakshman M.R., Lakshman M.R. (2010). Betaine Protects Chronic Alcohol and ω-3 PUFA-Mediated Down-Regulations of PON1 Gene, Serum PON1 and Homocysteine Thiolactonase Activities With Restoration of Liver GSH. Alcohol. Clin. Exp. Res..

[44] Goldstein E.S., Stephenson A., Gramstead G. Analysis of the chromosome region containing the Drosophila homolog of the jun oncogene. Proceedings of the 35th Annual Drosophila Research Conference.

